# Fitness Impaired Drug Resistant HIV-1 Is Not Compromised in Cell-to-Cell Transmission or Establishment of and Reactivation from Latency

**DOI:** 10.3390/v6093487

**Published:** 2014-09-19

**Authors:** Sophie M. Bastarache, Thibault Mesplède, Daniel A. Donahue, Richard D. Sloan, Mark A. Wainberg

**Affiliations:** 1 Department of Microbiology and Immunology, McGill University, Montreal, Québec H3T1E2, Canada; E-Mails: sophie.bastarache@gmail.com (S.M.B.); aaron.donahue@gmail.com (D.A.D.); 2 McGill University AIDS Centre, Lady Davis Institute for Medical Research, Jewish General Hospital, Montreal, Québec H3T1E2, Canada; E-Mails: tibo_mes@hotmail.com (T.M.); sloanrichard@gmail.com (R.D.S.)

**Keywords:** HIV-1, latency, transmission, integrase, drug-resistance

## Abstract

Both the presence of latently infected cells and cell-to-cell viral transmission are means whereby HIV can partially evade the inhibitory activities of antiretroviral drugs. The clinical use of a novel integrase inhibitor, dolutegravir (DTG), has established hope that this compound may limit HIV persistence, since no treatment-naïve patient treated with DTG has yet developed resistance against this drug, even though a R263K substitution in integrase confers low-level resistance to this drug in tissue culture. Here, we have studied the impact of R263K on HIV replication capacity and the ability of HIV to establish or be reactivated from latency and/or spread through cell-to-cell transmission. We affirm that DTG-resistant viruses have diminished capacity to replicate and establish infection. However, DTG-resistant viruses were efficiently transmitted via cell-to-cell contacts, and were as likely to establish and be reactivated from latent infection as wildtype viruses. Both cell-to-cell transmission of HIV and the establishment of and reemergence from latency are important for the establishment and maintenance of viral reservoirs. Since the DTG and other drug-resistant viruses studied here do not seem to have been impaired in regard to these activities, studies should be undertaken to characterize HIV reservoirs in patients who have been treated with DTG.

## 1. Introduction

Highly active antiretroviral therapy (HAART) has greatly improved both the quality of life and treatment outcomes for individuals diagnosed with HIV. However, there is still no cure for HIV infection and multiple obstacles remain before serious efforts aimed at HIV eradication in patients can be contemplated. These obstacles include HIV drug resistance, the efficiency of cell-to-cell viral transmission, and the establishment of latently infected cell reservoirs (reviewed in [1,2,3,4]). The problem of drug resistance necessitates the development of new antiretroviral (ARV) molecules that might prevent further viral transmission, both within a single patient and between individuals [5]. The most recent class of ARVs are integrase (IN) strand transfer inhibitors (INSTIs) that block integrase enzymatic activity by competitive inhibition [6,7].

The currently available INSTIs include raltegravir (RAL), elvitegravir (EVG) and dolutegravir (DTG). Both RAL and EVG possess moderate genetic barriers to the development of resistance [8], while DTG appears to be less susceptible to the emergence of drug resistance mutations [9]. Indeed, no resistance against either DTG or the compounds used together with it in treatment of previously drug-naïve individuals has ever been reported [10]. Moreover, the use of DTG to treat patients who had previously failed multiple drugs but who were naïve to INSTIs resulted in treatment failure in relatively few individuals, only two of whom developed the R263K mutation [11]. The latter substitution had previously been identified by our group on the basis of tissue culture selection experiments with DTG and was shown to diminish integrase enzymatic activity and viral DNA integration into host cells while conferring low-level resistance against DTG [12]. Further studies have shown that secondary mutations in integrase at positions H51Y and E138K that are associated with R263K failed to restore viral fitness [13,14]. DTG-resistant viruses were also unable to develop additional resistance mutations and were impaired in their ability to develop resistance against several reverse transcriptase (RT) inhibitors [15]. Altogether, these observations suggest that R263K may represent an evolutionary dead-end that could explain the scarcity of virological failures and resistance mutations in individuals treated with DTG [16].

HIV-1 can be transmitted between cells by either cell-free transmission or through direct contact between cells, *i.e.*, cell-to-cell transmission [17]. The latter, which results in the direct transmission of the virus from one cell to another through a virological synapse, is the primary mode of transmission both *in vitro* and in lymphoid tissues [18,19]; this allows coordinated viral assembly and viral entry, resulting in more efficient viral transmission between cells than occurs by cell-free transmission [20,21]. Infected cells are able to form polysynapses between one infected cell and multiple uninfected cells, which also increases the multiplicity of infection (MOI) of cell-to-cell transmission compared to cell-free transmission, whereby a single free virus particle can only infect one cell at a time [22,23,24]. Whether HAART is active against cell-to-cell transmission and what the relative importance is of this mode of transmission in the maintenance of the viral reservoir are still under debate [25,26,27,28]. Studies of cell-to-cell transmission of drug resistant viruses are warranted in order to determine the relationship between viral transmission, viral replicative fitness, and viral pathogenesis.

Similarly, it is important to study the latent HIV reservoir that is comprised of cells that house replication-competent proviruses that have been integrated into host chromosomal DNA. The fact that this latent population of viruses is not actively replicating means that it may be unaffected by current antiretroviral therapy and host immune defenses. However, appropriate stimulation causes latently infected cells to produce viral particles that can then infect other cells [29,30]. Both wildtype (WT) and drug-resistant viruses can be archived within the latent reservoir; thus, viral rebound due to either treatment interruption or failure can result in the production of any viral species that are present in the reservoir, allowing for the replication of drug-resistant viruses [31]. Since integrase inhibitors block strand-transfer activity, it is possible that mutations within integrase might lead to sites of preferential integration that could alter the potential of HIV to either establish latent infection or to achieve reactivation, a subject that is relevant to HIV cure research [32,33,34,35,36].

Here, we have asked whether DTG-resistance mutations might affect either the ability of HIV-1 to be transmitted or to establish latency. Our results show that DTG-resistant viruses can be efficiently spread through cell-to-cell transmission and can establish and be reactivated from latency as efficiently as WT virus, in spite of being impaired in regard to replication fitness.

## 2. Materials and Methods

### 2.1. Cell lines, Viruses, and Antiviral Compounds

Jurkat (clone E6-1) cells were obtained through the NIH AIDS Research and Reference Reagent Program and were maintained in RPMI 1640 medium (Invitrogen) supplemented with 10% fetal bovine serum (FBS), 1% L-glutamine, and 1% penicillin-streptomycin. pNL4-3-IRES-EGFP (expressing enhanced green fluorescent protein) was a kind gift from J. Munch and F. Kirchhoff [37,38]. The following constructs containing mutations in the integrase gene were created by site-directed mutagenesis: pNL4-3-IRES-EGFP-*IN*(R263K), pNL4-3-IRES-EGFP-*IN*(E138K), pNL4-3-IRES-EGFP-*IN*(E138K/R263K). The following construct containing a mutation in the reverse transcriptase (RT) gene was created by site directed mutagenesis: pNL4-3-IRES-EGFP-*RT*(M184V), as described previously [39]. Primers used for the generation of pNL4-3-IRES-EGFP-*IN*(R263K) have been previously reported [12]. The following primers were used for mutagenesis: *IN* E138K: sense: GGCGGGGATCAAGCAGAAATTTGGCATTCCCTA, antisense: TAGGGAATGCCAAATTTCTGCTTGATCCCCGCC. Replication-competent reporter viruses were produced by transfection of ~9 × 10^6^ 293T cells with 25 μg of plasmid DNA using Lipofectamine 2000 (Invitrogen). All transfections were carried out using Opti-MEM medium (Invitrogen) supplemented with 2.5% FBS. Virus-containing supernatants were harvested at 72 h post transfection, clarified by centrifugation for 5 min at 470× *g*, and passed through a 0.45-μm-pore filter. All viruses were then treated with 50 U/mL benzonase (Sigma) in the presence of benzonase buffer (50 mM Tris-HCl [pH 8.0], 1 mM MgCl_2_, and 0.1 mg/mL bovine serum albumin [BSA]) at 37 °C for 20 min to digest residual plasmid DNA. Viral titers were determined by enzyme linked immunosorbtion assay (ELISA) for viral capsid (p24), using a Vironostika HIV-1 antigen (Ag) kit (BioMérieux). The protease (PR) inhibitor darunavir (DRV) was obtained through the National Institutes of Health AIDS Research and Reference Reagent Program.

### 2.2. Infectivity Assay in TZM-bl Cells

Relative infectivity of the recombinant WT and mutant viruses was measured using a non-competitive short-term infectivity assay in TZM-bl cells as described previously [12]. Thirty-thousand cells were seeded into 96-well culture plates and infected for 48 h with the indicated virus. Luciferase was measured using a Luciferase assay system kit (Promega) and a MicroBeta2 Luminometer (Perkin Elmer). GraphPad Prism 5.0 software was used to assess relative viral replication capacity based on measurement of viral RT activity in culture media.

### 2.3. Jurkat Cell Latency Model

Populations of latently infected cells were established as described previously [38], with some modifications. Briefly, untreated Jurkat cells were infected through spinoculation with NL4-3-IRES-EGFP, NL4-3-IRES-EGFP-*IN*(R263K), NL4-3-IRES-EGFP-*IN*(E138K), NL4-3-IRES-EGFP-*IN*(E138K/R263K), and NL4-3-IRES-EGFP-*RT*(M184V) cell-free viral particles, using 600 ng p24 per million cells for 2 h at 1200 g, after which cells were washed twice with PBS and cultured for 7 days in the presence of 1 μM DRV to ensure single-round replication. After 7 days, samples were treated for 24 h with tumor necrosis factor α (TNF-α) (20 ng/mL) to reactivate latent viruses, before being fixed in 2% paraformaldehyde (PFA) for 20 min. Flow cytometry was performed using a LSR Fortessa cell analyzer (Becton Dickinson) and data were analyzed with FlowJo software. Live cells were gated by forward and side scatter properties; single cells were then gated based on forward and side scatter width and height and levels of EGFP were then measured.

### 2.4. Cell-to-Cell Transmission Assay

Cell-to-cell transmission assay was performed as previously described, with some modifications [40]. Briefly, untreated Jurkat cells were infected through spinoculation with NL4-3-IRES-EGFP, NL4-3-IRES-EGFP-*IN*(R263K), NL4-3-IRES-EGFP-*IN*(E138K), NL4-3-IRES-EGFP-*IN*(E138K/R263K), and NL4-3-IRES-EGFP-*RT*(M184V) cell-free viral particles, using 600 ng p24 per million cells for 2 h at 1200 g, after which cells were washed twice with PBS and cultured for 72 h. After 72 h, one-quarter of the infected sample were fixed with 2% paraformaldehyde for 20 min, and the other three-quarters of the sample (donor cells) were co-cultured with uninfected Jurkat target cells stained with CellTrack Violet (Invitrogen) at a ratio of 2:1 infected:uninfected cells for 24 h or 72 h to measure cell-to-cell transmission from the infected cells to the uninfected cells. When cell-to-cell contacts were prevented through the use of Transwell chambers (Corning) or when culture fluids only were transferred to the uninfected Jurkat target cells (cell-free transmission), the infection of target cells was undetectable (<0.01%) using the method described below. For the 72 h co-culture only, 1 μM DRV was added at the beginning of co-culture to ensure single-round infection. Following co-culture, samples were fixed with 2% paraformaldehyde for 20 min. Flow cytometry was performed using a LSR Fortessa cell analyzer (Becton Dickinson) and data were analyzed with FlowJo software. Live cells were gated by forward and side scatter properties, and single cells were then gated based on forward and side scatter width and height. Viruses transmitted via cell-to-cell contact were selected for by gating the CellTrack Violet-stained target cell population, and levels of EGFP were then measured.

### 2.5. Statistical Analyses

One-way analysis of variance (ANOVA) and Dunnett’s Multiple Comparison tests were performed using GraphPad Prism 5.0 software.

## 3. Results

### 3.1. The Drug-Resistant Viruses Tested Are Impaired in Viral Replication

First, we quantified infectivity using a TZM-bl away and measuring levels of RT that were produced following infection (Table 1). The results show that the R263K substitution decreased HIV-1 infectivity, as did the E138K and both the E138K/R263K mutations in tandem that resulted in significant decrease in RT activity. As a control, we also studied M184V-containing 3TC-resistant viruses and also showed that it results in lower RT levels, in agreement with previous studies [41].

**Table 1:** Effect of INSTI-resistance mutations on the relative HIV-1 replication capacity based on reverse transcriptase activity in TZM-bl cells.

Backbone	Genotype of Mutation	RT Activity (% of WT)	95% Confidence Intervals
NL4.3	WT	100	89 to 113
	M184V (RT)	77	68 to 88
	E138K (IN)	46	42 to 51
	R263K (IN)	42	38 to 46
	E138K/R263K (IN)	37	34 to 40

### 3.2. Drug-Resistant Viruses Are as Effective at Cell-to-Cell Transmission as WT Viruses

Next, we examined the efficiency of HIV cell-to-cell transmission *vs.* cell-free transmission using DTG-resistant viruses that contained either the R263K, E138K or E138K/R263K mutations [12]. We used also a 3TC/FTC-resistant virus containing a M184V mutation in the RT gene as a control [42,43].

**Figure 1:**
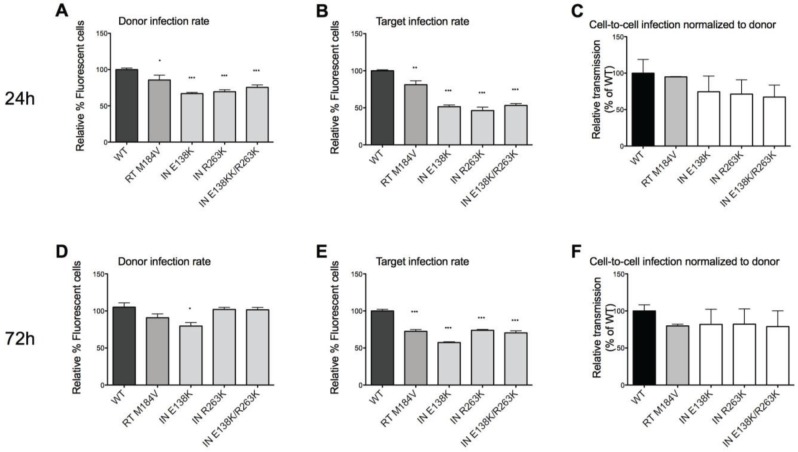
Cell-to-cell transmission by WT and drug-resistant viruses. (**A**,**D**) Relative reporter virus GFP expression of infected donor cells prior to co-culture for 24 h or 72 h respectively, compared to WT. Mutant viruses were less infectious than WT. (**B**,**E**) Relative reporter virus GFP expression of infected target cells after co-culture for 24 h or 72 h, respectively, compared to WT. Fewer target cells were infected with the mutant viruses than with WT. (**C**,**F**) Relative proportion of cells infected via cell-to-cell transmission by measurement of GFP expression by reporter virus after 24- or 72-h co-culture, respectively. No statistical differences in the rates of cell-to-cell transmission were observed between the different viruses. Relative expression of GFP in target cells compared to WT after the co-culture was assessed on the basis of relative expression of GFP in donor cells compared to WT prior to co-culture. Statistically significant differences between drug-resistant mutated viruses and WT viruses are indicated. The absence of an asterix indicates no statistical difference from WT. Error bars represent standard error of the mean (SEM, four independent experiments were performed for 24 h co-culture, two independent experiments were performed for 72 h co-culture. All experiments were performed in triplicate).

Quantitation of infectivity with an assay that measured GFP expression in donor cells showed that the drug-resistant viruses had moderately lower infection efficiency than WT viruses, corresponding the results of Table 1 (Figure 1A). Using trans-well chambers that inhibit direct cell-cell contacts, we have previously shown the inefficiency of cell-free viral transmission in this system [40]. We can, thus, conclude that any GFP detected in the target cells after co-culture is the result of cell-to-cell transmission. We also found that cell-to-cell transmission of the various mutated viruses that were tested was slightly decreased compared to WT but that the differences were insignificant after 24 h co-culture (Figure 1B,C). Co-culture of donor and target cells for 72 h yielded similar results (Figure 1D–F), with slightly higher cell-to-cell transmission rates than after 24 h, since there was more time for viral spread. The modest decreases in infectiousness of INSTI-resistant viruses are in agreement with the results of the diminished replication fitness of the INSTI-resistant viruses.

### 3.3. Drug-Resistant Viruses Are as Able to Establish Latent Infection as WT Viruses

**Figure 2:**
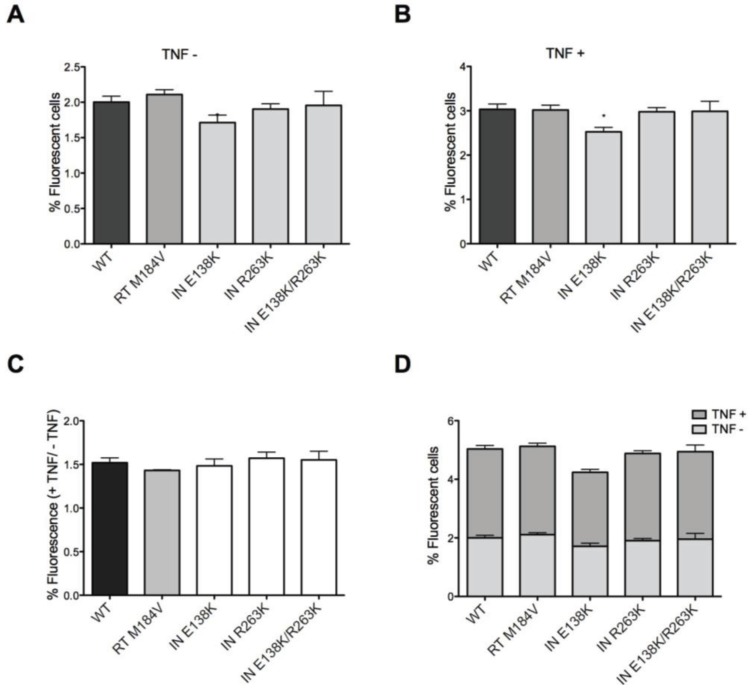
Establishment of and reactivation from latency by WT and drug-resistant viruses. (**A**) Expression of GFP reporter virus in Jurkat cells cultured in the presence of DRV after 7-days of infection. GFP expression in this experiment represents the background of actively replicating viruses that have not achieved latency. No statistically significant differences were observed between the various viruses; (**B**) Expression of GFP reporter virus in Jurkat cells grown in the presence of DRV for 7-days after infection, following overnight TNFα treatment to reactivate latent proviruses. DRV was used for the purpose of ensuring that only a single round of infection would occur. Only E138K resulted in a decrease in the proportion of infected cells (*); (**C**) Relative proportion of GFP-expressing reporter proviruses that became reactivated following TNFα treatment. The results show the relative GFP expression of reporter virus after TNFα treatment divided by relative GFP expression of reporter virus before TNFα treatment. No statistically significant differences were observed between the various viruses; (**D**) Comparison of TNFα-treated samples and untreated samples for each virus tested. No statistically significant differences were observed between the various viruses. Statistically significant results for drug-resistant mutated viruses compared with the WT control are indicated. Error bars represent standard error of the mean (SEM, two independent experiments, performed in triplicate).

To determine whether drug resistance mutations in IN might affect the establishment of and reactivation from latency, we used a modified version of a previously described Jurkat model [38]. As shown above, mutations within IN were shown to result in insignificant decreases in infectivity compared to WT over a period of seven days, this time using an assay that measures number of infected cells, based on GFP reactivity with the Nef protein (Figure 2A). After seven days of infection, TNF-α was used to treat the infected cells to cause viral reactivation from potential latency, as described in Materials and Methods. The results show that the treatment resulted in a ~50% increase in the number of GFP-positive cells compared to non-treatment (Figure 2B). The presence of E138K mutation alone caused only slight decreases in GFP expression, both before and after TNF-α treatment, and the combination of E138K and R263K mutations together had no additional effect on GFP expression (Figure 2B). These trends in GFP expression are consistent with the data on cell-to-cell transmission presented above (Figure 1).

Figure 2C confirms these observations on the basis of the ratios between levels of GFP expression between infected cells that were treated or not with TNF-α. Furthermore, Figure 2D presents a direct comparison of the treated *vs.* untreated cells in terms of GFP expression.

Thus, the INSTI-resistant viruses seem to be as capable of establishing latent infection as WT viruses. Any differences in viral expression before or after TNF-α treatment may simply be due to differences in relative viral fitness and infectiousness of the viruses tested.

### 3.4. Viral Reactivation Determined by Flow Cytometry

**Figure 3:**
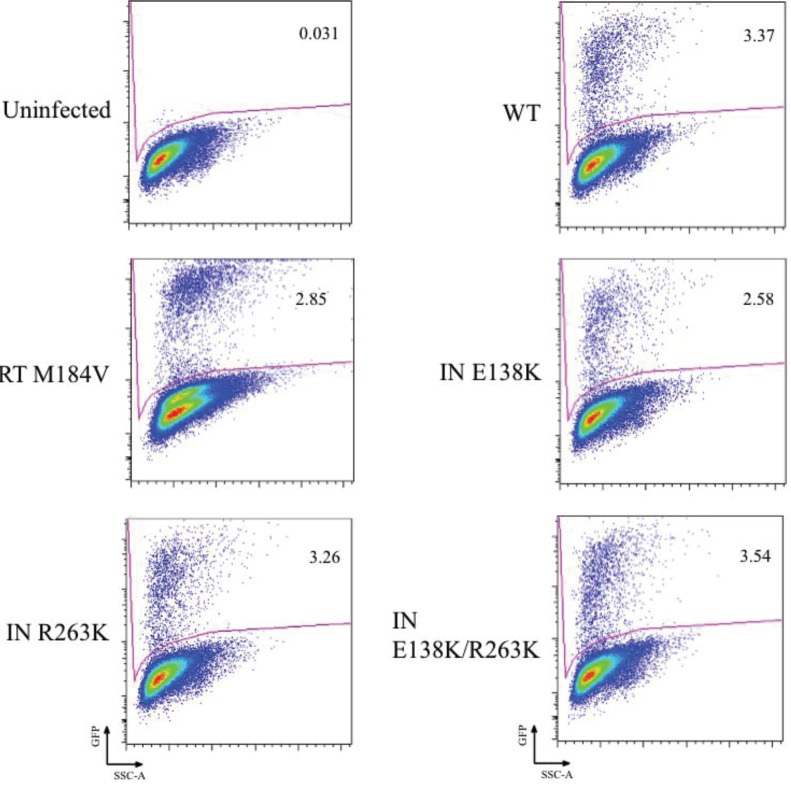
Reactivation of latently infected Jurkat cells. Jurkat cells that were either infected or uninfected with WT or INSTI-resistant viruses were cultured for 7 days in 1 μM DRV to inhibit new infections before reactivation with 20 ng/mL TNF-α. Representative results are shown here. As shown in Figure 2, there were no statistical differences between the mutants and WT viruses. Gating strategy is shown, and the percent of reactivated virus is denoted within the gate. SSC-A, side scatter area. GFP represents activated virus.

The results of representative studies in which viruses were reactivated from latency by treatment with TNF-α are shown in Figure 3. The percentage of cells that have expressed reactivated virus, based on GFP expression, is shown within the gate for each of the viruses that were studied. In each case, the percentage of cells expressing GFP is approximately 50% higher than for untreated cells.

## 4. Discussion

We have hypothesized that the R263K mutation in HIV integrase may contribute to the prevention of virological failure in patients who are treated with DTG in combination with other ARVs [16]. Since cell-to-cell transmission and the establishment of latency reservoirs are two major obstacles to HIV eradication, we measured how DTG-resistance might impact these two activities. Here, we have confirmed the decrease in fitness of DTG-resistant viruses by demonstrating reduced infection efficiencies, as measured in both TZM-bl cells (Table 1) and by the percent of GFP-expressing cells (Figure 1).

We noted that the mutated viruses, in general, shared the same capacity for cell-to-cell transmission as WT viruses (Figure 1B,E). We hypothesize that drug-resistance mutations have little effect on either cell-free transmission or transmission via virological synapses. Cell-to-cell transmission is highly efficient [18,44]; if a mutation were to lead to increased facility of transmission, this could result in increased challenges for treatment. Although this does not seem to happen, it is also significant that viruses that are resistant to RT and protease inhibitors can spread between cells as efficiently as WT viruses [25,28]. Ours is the first study to demonstrate this in integrase-resistant viruses.

Similar results were obtained in regard to the establishment of and reactivation from latency. After seven days of infection, low-level background expression of virus remained, as determined by GFP fluorescence and flow cytometry, probably due to residual active infection (Figure 2A and Figure 3). When these basal active infections are taken into account, all viruses, including drug-resistant mutants and WT, showed an approximate 50% increase in virus expression following TNF-α treatment (Figure 2C). This demonstrates that the ability of virus to establish latency depends on both fitness and infectiousness and that all the viruses tested possessed similar ability to establish and be reactivated from latency. This conclusion is supported by the fact that M184V-containing virus for which viral replication capacity is also diminished compared to WT also sustained similar levels of cell-to-cell transmission as well as establishment of and reactivation from latency.

At present, researchers are developing ways to reactivate expression of latent virus in order to purge HIV reservoirs [35,36]. As shown here and elsewhere, both WT and drug resistant viruses can establish latency, and reactivation will doubtless result in infection of both uninfected and previously infected cells, leading to possible recombination in the latter situation [31]. We have previously reported that cells can be reactivated by superinfection and that recombination can result in the emergence of multi-drug resistant variants, if both the latent and superinfecting viruses contain drug resistance mutations [45].

The findings presented here show that DTG-resistant viruses are not impaired in their ability to participate in cell-to-cell transmission or to establish and/or reemerge from latency, despite the fact that the mutated viruses that we studied were diminished in replication capacity. Although cell-to-cell transmission and the establishment of latency and reactivation from it are not impaired in DTG-resistant viruses, it will still be important to investigate whether DTG-treatment and/or drug resistance impacts on the size and quality of viral reservoirs, including those in patients who are successfully treated with DTG or who may ultimately develop resistance against this drug.
